# Marine-Derived Terpenes: Chemistry, Synthesis and Their Therapeutic Potential

**DOI:** 10.3390/md23120483

**Published:** 2025-12-17

**Authors:** Jinmei Xia

**Affiliations:** 1 Key Laboratory of Marine Biogenetic Resources, Third Institute of Oceanograhy, Ministry of Natural Resources, Xiamen 361005, China; xiajinmei@tio.org.cn; 2 State Key Laboratory of Natural and Biomimetic Drugs, Peking University, Beijing 100191, China

The past five years have marked a significant evolution in terpenoid natural product research, with direct implications for marine drug discovery. Innovations such as high-yield engineered microbial hosts, automated high-throughput reconstruction of biosynthetic gene clusters, artificial intelligence-guided enzyme identification, and combinatorial pairing of terpene synthases with tailoring enzymes have collectively addressed longstanding issues of redundant discoveries, minimal yields, and constrained structural variety [1]. Initially optimized in terrestrial fungal and bacterial systems, these platform technologies are now being adapted to marine-derived microorganisms and associated microbial communities, greatly enhancing access to previously cryptic or under-expressed terpene gene clusters from sponges, corals, tunicates, deep-sea sediments, and polar ecosystems [2,3].

Despite ongoing progress, research on marine terpenoids continues to face several critical challenges. There remains a lack of efficient and systematic discovery pipelines for lead compounds, and the exploration of novel, highly active terpene structures is still limited. Much of the research continues to rely on preliminary in vitro screening, lacking in-depth mechanistic elucidation or in vivo validation, which hinders translation into targeted therapeutic strategies. Furthermore, a comprehensive understanding of the broader chemical diversity and biosynthetic logic of these compounds remains insufficient, making it difficult to systematically harness the potential of marine extracts for the development of treatments against major diseases such as drug-resistant cancers and infections.

The five manuscripts in this Special Issue together make a significant contribution and greatly expand our understanding in the field of chemical and pharmacological research on marine terpenoids.

Contribution 1: http://doi.org/10.3390/md23020078.

Heo et al. reported the discovery of four new kaurane-type diterpenoids, geliboluols A–D, isolated from the marine-derived rare actinomycete *Actinomadura geliboluensis*. The cytotoxic potential of the compounds was evaluated against a panel of cancer cell lines. Notably, geliboluol D exhibited moderate cytotoxic activity. This study highlighted marine actinomycetes as a valuable and underexplored source of structurally novel diterpenes.

Contribution 2: http://doi.org/10.3390/md22060274.

Tang et al. obtained three new pentaketide-sesquiterpenes, 8,9-*epi*-chrodrimanins, from the marine-derived fungus *Talaromyces variabilis*. They evaluated the biological activity of these compounds and identified one as a selectively cytotoxic agent against MKN-45 gastric cancer cells. This finding underscores the therapeutic potential of marine terpenes and demonstrates how subtle structural changes can dramatically influence bioactivity.

Contribution 3: http://doi.org/10.3390/md22060245.

Wang et al. established a robust UHPLC-QTOF MS screening platform to discover citrate lyase inhibitors from a custom marine compound library. Their work successfully identified a substantial portion of marine-derived terpenoids as potent inhibitors of this enzyme, which is crucial for *Mycobacterium tuberculosis* survival. This study not only provides an efficient methodological approach for inhibitor discovery but also directly expands the known therapeutic potential of marine terpenoids into the critical area of anti-tuberculosis drug development.

Contribution 4: http://doi.org/10.3390/md23040171.

Cheng et al. systematically summarized recent advances in the antiparasitic effects of seaweed extracts. The review compellingly argues for marine algae as a rich and underexplored source of novel therapeutic agents against major parasites like *Plasmodium*, *Leishmania*, and *Trypanosoma*. It highlights the role of terpenoids, among other unique marine compounds. By providing a foundational framework and emphasizing the potential to overcome growing drug resistance, this work effectively charts a course for future drug discovery efforts rooted in marine biodiversity.

Contribution 5: http://doi.org/10.3390/md23020072.

Zhang et al. provided a timely and systematic overview of 447 marine-derived diterpenes discovered between 2019 and 2024. They classified these compounds based on their skeletal structures and detailed their diverse biological activities, including antiviral, anti-inflammatory, and anticancer properties. The review also featured modern structural elucidation techniques and explored synthetic strategies for key compounds. The article offered an invaluable resource that underscores the structural richness and therapeutic potential of marine diterpenes.

In summary, these contributions signify a field progressing from isolated structure elucidations toward cohesive discovery platforms aligned with contemporary pharmaceutical standards.

Projecting into the next decade, the integration of approaches outlined in recent pivotal studies provides a feasible roadmap for advancing marine terpenoid leads to clinical stages. Multi-omics-driven genome mining of uncultivated sponge, coral, and deep-sea microbiomes, coupled with efficient heterologous expression in refined yeast and filamentous fungal hosts, will activate silent clusters on a massive scale. Artificial intelligence and AlphaFold-based protein modeling, as exemplified by the identification of non-squalene triterpene cyclases [4] and the engineering of redox-enhanced *E. coli* platforms generating hundreds of novel diterpenoids in one run [5], will hasten the discovery and modification of new terpene synthases, prenyltransferases, and modifying enzymes from marine origins. Automated synthetic biology workstations connected to enzymatic and cellular screening pipelines will produce extensive structure–activity relationship datasets, enabling robust machine-learning predictions of bioactivity and pharmacokinetics. Moreover, embedding preclinical absorption, distribution, metabolism, excretion, and toxicity assessments, along with target validation and animal efficacy testing, early in optimization workflows will narrow the divide between promising in vitro candidates and viable drug prospects.

By applying these integrated technologies systematically to the vast phylogenetic and ecological richness of marine microorganisms and symbionts, the oceans’ potential as a prolific source of terpenoid therapeutics is on the cusp of fulfillment. The works featured in this Special Issue offer both the chemical blueprints and methodological groundwork to achieve that goal.
